# Chikungunya: an emerging viral infection with varied clinical presentations in Bangladesh: Reports of seven cases

**DOI:** 10.1186/s13104-017-2723-5

**Published:** 2017-08-15

**Authors:** Muhammad Abdur Rahim, Khwaja Nazim Uddin

**Affiliations:** Bangladesh Institute of Research and Rehabilitation in Diabetes, Endocrine and Metabolic Disorders (BIRDEM) General Hospital, 122 Kazi Nazrul Islam Avenue, Dhaka, Bangladesh

**Keywords:** Bangladesh, Chikungunya, Dhaka, Dengue, Outbreak

## Abstract

**Background:**

Chikungunya is an emerging and rapidly spreading viral infection in many parts of the world including Bangladesh. It shares many epidemiological and clinical characteristics with dengue. So, a sound knowledge is required for its detection and differentiation from dengue, specially in endemic regions.

**Case presentation:**

We present seven confirmed cases of chikungunya having different clinical presentations occurring among middle aged males and females from different socio-economic background in Dhaka city, the capital of Bangladesh. All patients had fever and aches and pains. Less common features were rash, diarrhea, vomiting and altered liver biochemistry. Dengue was excluded in six patients. Paracetamol remained the mainstay of treatment during febrile periods, but over 50% of the patients had prolonged joint symptoms requiring non-steroidal anti-inflammatory drugs.

**Conclusions:**

In spite of being a self-limiting disease, chikungunya may have different presentations and a protracted clinical course. During the febrile episode, exclusion of dengue is equally important. Physicians should be aware of the condition and public health initiatives are necessary to break the disease transmission.

## Background

Chikungunya is one of the most rapidly spreading mosquito-borne viral infectious diseases [1] and recently in Bangladesh it has emerged as an important public health issue [2]. Over the previous years, infrequent outbreaks [3, 4] and only a few cases of chikungunya [5] were reported from Bangladesh. Generally, it is a self-limiting disease with fever and arthralgia/arthritis being the two most common features; though it may have diverse presentations [1] and fatal outcomes are rarely reported [6]. Here, we report seven autochthonous cases of chikungunya having varied clinical presentations among adult Bangladeshi patients.

## Case presentation

### Case 1

A 40-year-old physician got admitted with a 2-day history of high grade continued fever and severe generalized aches and pains involving trunk, hands, feet and spine. His fever peaked up to 104 °F. His pain was so severe that he could not move from side to side in bed or sit without help of others in first 2–3 days. Fever did not touch base-line in first 2 days with paracetamol and sponging. He vomited several times on day of admission. He did not have any retro-orbital pain and he denied any recent travel history.

On admission, the patient was febrile (temp 104 °F), tachycardic (pulse 112/min) and dehydrated. There was no rash, lymphadenopathy or meningism. Other systemic examination findings were normal.

His complete blood count (CBC) revealed lymphopaenia (Table 1). Dengue nonstructural protein 1 (NS 1) [done by immunochromatographic test (ICT) by using commercially available kits manufactered by Humasis Co. Ltd., Republic of Korea] was negative. A blood film did not reveal any malarial parasite.

**Table 1:** Summary of findings of cases of chikungunya (N=7)

Findings	Case 1	Case 2	Case 3	Case 4	Case 5	Case 6	Case 7
Age (years)	40	37	35	38	30	41	52
Sex	Male	Female	Male	Female	Male	Female	Male
Occupation	Physician	Service holder	Businessman	Housewife	Unemployed	Housewife	Service holder
Fever at presentation (days)	2	4 (1 week back)	1	4	2-3	1	1
Total duration of fever (days)	5	4	4	5	5	4	5
Arthralgia/arthritis	Yes, hands, feet	No	Yes, left knee	No	Yes, wrists, hands, feet	Yes, wrists, hands, ankles	Yes, left ankle
Rash	Yes, on day 6	Yes, on day 1	No	No	No	No	No
Lymphadenopathy	Yes, post-auricular	No	No	No	No	No	No
Others	Vomiting	–	–	–	Loose motion	–	Vomiting Loose motion
Hb (gm/dl)	11.8	13.5	13.9	11.9	9.4	11.3	12.2
Hct%	34.9	35.1	35.3	34.6	29.5	35.2	35.8
TC of WBC (X10^9^/L)	6.68	4.0	6.13	5.78	10.55	7.23	7.22
Lymphocytes (%)	8.2	38	20.1	34	9.5	21.3	10
Platelets (X10^9^/L)	223	230	197	234	188	334	191
ESR (mm/1^st^ hr)	7	27	12	34	65	46	28
CRP (mg/L)	25.9	–	–	–	–	–	34
NS 1	Negative	–	Negative	Negative	Negative	Negative	Negative
Bilirubin (mg/dl)	0.8	–	–	–	0.4	–	0.7
ALT (U/L)	15	–	–	–	254	–	48
AST (U/L)	20	–	–	–	81	–	56
CPK (U/L)	51	–	–	–	–	–	61
S.Creatinine(mg/dl)	1.0	–	–	–	11.1	–	0.9
Electrolytes, mmol/L	Na–K–Cl–TCO_2_ 139–3.9–105–26	–	–	–	Na–K–Cl–TCO_2_ 136–5.5–102–25	–	Na–K–Cl–TCO_2_ 135–3.6–102–21
Blood culture	No growth	–	–	–	No growth	–	No growth
RT-PCR for chikungunya	–	Positive	–	–	–	–	–
Anti-chikungynya antibody (IgM) ICT	Positive	–	Positive	Positive	Positive	Positive	Positive
Anti-chikungynya antibody (IgG)	Negative	–	Negative	Negative	Negative	Negative	Negative
Residual joint symptom (beyond febrile illness)	Yes, left foot	No	Yes, right ankle	No	No	Yes, right ankle	Yes, left ankle

Treatment consisted of paracetamol and anti-emetics along with supportive measures like intravenous fluids in first 2 days.

He was discharged after 4 days of admission in an afebrile state with a diagnosis of viral fever. One day later he developed loose motion, 4–5 times, containing foul smelling stools without any blood or accompanying abdominal pain. He noticed painful lymphadenopathy involving bilateral post-auricular group on day 6 post-discharge and maculo-papular rash involving mostly hands and palms. Repeat CBC was normal. Considering chikungunya as a possibility, anti-chikungunya immunoglobulin M (IgM) (done by ICT for chikungunya IgM/IgG by using commercially available kits manufactured by SD BIOSENSOR, Republic of Korea) was done and it appeared positive. He became active by day 10 with pain in left foot requiring naproxen 500 mg twice daily.

### Case 2

A 37-year-old office executive presented with a report of positive chikungunya IgM (ICT, SD BIOSENSOR, Republic of Korea) antibody report. She suffered an acute febrile illness of 4-days duration 1 week ago. She had severe generalized body ache and rash on the very first day of her ailment. She did not have any definite joint pain during fever or thereafter. She received paracetamol and became afebrile on day 4. For confirmation of her diagnosis, she underwent the test as per her general practitioner’s advice and found it to be positive (Table 1).

### Case 3

A 35-year-old small scale self-employed businessman presented with 1-day history of fever along with pain and swelling of left knee joint. He was febrile with a temperature of 103 °F, tachycardic and had normal blood pressure. His left knee joint was swollen and tender. He did not have recent history of diarrhea or high risk sexual activity. Systemic examination was unremarkable. He had normal haemogram and dengue NS 1 was negative but he tested positive for reverse transcriptase-polymerase chain reaction (RT-PCR) for chikungunya (Qualitative One-Step Real-Time Reverse-Transcriptase PCR technology in the ABI 7500 DX instrument with SDS software) (Table 1). He was initially treated with paracetamol, later he required ibuprofen for pain relief. In spite of initial satisfactory improvement, he reported back after 3 weeks with pain and swelling of right ankle joint. He was reassured and ibuprofen was prescribed. He became symptom free after 5 days.

### Case 4

A 38-year-old house wife presented with 4-day history of fever and generalized aches and pains. She did not have any retro-orbital pain. Her blood counts were normal (Table 1). She was on paracetamol. She was advised to undergo anti-chikungunya antibody test after 3 days and it became positive (ICT, SD BIOSENSOR, Republic of Korea) (Table 1); by that time she recovered completely without any residual articular symptom.

### Case 5

A 30-year-old male, diagnosed with type 1 diabetes mellitus and end stage renal disease on maintenance haemodialysis got admitted with a 3-day history of fever and pain involving wrists, hand joints and joints of the feet. He also complained of loose motion and vomiting for the same duration. He was haemodynamically stable. He had lymphopaenia (Table 1) and dengue NS 1 (ICT, Humasis Co. Ltd., Republic of Korea) was negative. He tested positive for anti-chikungunya antibody IgM (ICT, SD BIOSENSOR, Republic of Korea) after 5 days of admission. He was treated with paracetamol and anti-emetics and his usual treatment including haemodialysis was continued.

### Case 6

A 41-year-old housewife presented with a 1-day history of fever and arthritis involving wrists, hands and ankles. She had normal blood counts and negative NS 1 for dengue (ICT, Humaisi Co. Ltd., Republic of Korea) and subsequent blood test confirmed her as having chikungunya (Table 1). She recovered with paracetamol, but experiencing persistent pain in right ankle requiring ibuprofen.

### Case 7

A 52-year-old hospital staff got admitted with 1-day history of fever, left ankle pain and vomiting. He had lymphopaenia and high erythrocyte sedimentation rate (ESR) (Table 1) and negative NS 1 (ICT, Humasis Co. Ltd., Republic of Korea). He was discharged after 4 days in afebrile state. During his follow-up visit after 1 week, he came with a positive anti-chikungunya IgM (ICT, SD BIOSENSOR, Republic of Korea) test report and was continuing with pain and swelling in left ankle joint. He was put on ibuprofen with improvement of symptoms.

## Discussion

Since the first outbreak of chikungunya virus in 1952–53 in Tanzania [7, 8], several outbreaks of chikungunya had occurred in different countries [9]. In Bangladesh two outbreaks were reported in 2008 and in 2011 [3, 4]. In previous years, outbreaks had occurred in India [10, 11], Pakistan [12, 13], Thailand [14] and Brazil [15, 16].

Chikungunya is caused by a vector-borne virus of genous Alphavirus and family Togavirus. *Aedes aegypti* and *Aedes albopictus* are the usual vectors, which are also responsible for transmission of dengue, hence well explains concurrence or co-incidences of dengue and chikungunya in endemic regions [17]. In Bangladesh, over the past two decades, dengue had been recognized as endemic disease with high incidence during the rainy seasons especially in big cities. This year’s outbreak of chikungunya also parallels the same seasonal and environmental characteristics of dengue in Dhaka, Bangladesh. *Aedes albopictus* [4] was the main vector in 2011 outbreak in Dohar, Dhaka. Scientists forecasted chikungunya as an emerging viral infection in Bangladesh in 2014 as well [5]. A National Guideline has been prepared for clinical management of chikungunya fever in Bangladesh [18].

Generally, chikungunya is a self-limiting viral infection with fever, arthritis and rash. Some patients may have protracted course of joint symptoms [19] meriting rheumatological investigations. Besides these, chikungunya can involve various body systems [1, 20] including central nervous system [21] and cardiovascular system [22] and rarely may be fatal one, especially in non-endemic regions and delay in diagnosis due to non-familiarity of the condition may be the reason behind [6].

Our patients had fever, joint pain and rash. Three patients had gastrointestinal symptoms and one patient developed lymphadenopathy. Four of our patients had persistent/prolonged joint symptoms requiring non-steroidal anti-inflammatory drugs (NSAIDs). We did not find any neurological or cardiovascular involvements but 2 patients had abnormal liver biochemistry (Table 1, Case 5 and 7) that resolved subsequently. Clinical features and seasonal similarities make it difficult to distinguish from dengue. Absence of retro-orbital pain, neutropaenia and thrombocytopaenia, negative NS 1 and presence of rash at onset of febrile illness, lymphopenia [1], high ESR and C-reactive protein (CRP) are important clues in differentiation between the two. Exclusion of differentials are sometimes more important than establishing a diagnosis of chikungunya. We did not face any zika infection in our clinical practice, which is an important differential of chikungunya.

During an outbreak, clinical and epidemiological criteria may suffice to make a probable diagnosis of chikungunya fever, but confirmation requires fulfillment of laboratory critera irrespective of clinical presentation [18]. RT-PCR may be done in first week and IgM may appear after day 5 of symptom onset and may last for weeks to months [1]. Raised ESR and presence of lymphopaenia are important in laboratory differentiation from dengue. If patient comes early, an NS 1 may be done to exclude dengue.

Treatment is aimed at relieving symptoms with paracetamol. Once dengue is excluded, NSAIDs may be used for severe joint symptoms. Four of our seven patients required NSAIDs. Prevention of disease transmission by confining the patient using mosquito nets through-out the disease course is one of the important interventions [1, 18]. Clearing house-holds and mosquito breeding sites can give double benefit against chikungunya and dengue [1, 18, 23].

## Conclusion

Chikungunya is a relatively new entity in Bangladesh. During the rainy season, an acute febrile illness with joint pain should raise the suspicion, but important is to exclude dengue rather than establishing the diagnosis of chikungunya. For prevention, public health initiatives are warranted.

## Abbreviations

CBC: complete blood count
NS 1: nonstructural protein 1
IgM: immunoglobin M
RT-PCR: reverse transcriptase-polymerase chain reaction
ESR: erythrocyte sedimentation rate
CRP: C-reactive protein
NSAIDs: non-steroidal anti-inflammatory drugs
